# Cervical myelopathy caused by IgG4-related hypertrophic spinal pachymeningitis: Case report and a descriptive review of the literature

**DOI:** 10.1016/j.bas.2024.103325

**Published:** 2024-09-10

**Authors:** Ahmet Parlak, Christian-Andreas Mueller, Kay W. Nolte, Tobias P. Schmidt, Ulf Bertram, Hans Clusmann, Christian Blume

**Affiliations:** a Department of Neurosurgery, RWTH Aachen University, Pauwelsstrasse 30, 52074, Aachen, Germany; b Institute of Neuropathology, RWTH Aachen University, Pauwelsstrasse 30, 52074, Aachen, Germany

**Keywords:** Immunoglobulin G4 (IgG4), Immunoglobulin G4-related disease (IgG4-RD), Immunoglobulin G4-related hypertrophic pachymeningitis (IgG4-RHP), Hypertrophic spinal pachymeningitis, Myelopathy

## Abstract

**Introduction:**

IgG4-related disease is an immune-mediated condition characterized by tissue infiltration of IgG4-positive plasma cells. Involvement of the spinal meninges results in hypertrophic spinal pachymeningitis (HSP), causing spinal cord and nerve root compression.

**Research question and case description:**

In this review, we present a case of IgG4-related hypertrophic spinal pachymeningitis. Furthermore, we provide an updated literature review on IgG4-related HSP.

**Materials and methods:**

We describe the case of a 45-year-old male presenting with cervical myelopathy. MR-imaging showed a ventrodorsal thickening of the meninges resulting in spinal cord compression. The patient underwent surgical decompression through laminectomy and excision of the dural thickening. The pathological findings demonstrated hypertrophic pachymeningitis with further examination showing large-scale dural infiltration of IgG4-positive lymphocytes. Adjuvant therapy with methylprednisolone and rituximab resulted in full neurological recovery with no signs of recurrence on MRI or clinically 12 months postoperatively.

An updated review of the literature regarding IgG4-related HSP was performed according to PRISMA-guidelines. Relevant articles were searched from the PubMed, Web of Science and Embase databases. Patient characteristics, MRI- and histopathological findings, treatment modality and outcome were reviewed.

**Results:**

The literature review provided a summary of 52 available cases, which included the one cases from our centre. Progressive worsening of neurological impairment was observed in 28 patients (58%). The lesions involved the thoracic spine (n = 33, 62.2%), cervical spine (n = 35, 70%), lumbar spine (n = 10, 20%), and sacral spine (n = 1, 2.2%). The dural thickening typically appeared as striated, fusiform, or oval changes, with homogeneous and patterns being the most common. Surgical decompression followed by immunosuppressive treatment was the main choice of therapy. The disease proved fatal in one case.

**Discussion and conclusion:**

IgG4-related HSP usually affects the cervical and thoracic dura and therefore often presents with myelopathy. Surgical decompression in cases of neurological deficits may prevent permanent neurological impairment. Immunosuppressive therapy constitutes the cornerstone in the treatment IgG4-related HSP.

## Highlights

• IgG4-related disease can involve the spinal meninges causing hypertrophic spinal pachymeningitis.
• Myelopathy is the most common clinical presentation.
• The thoracic spine is most involved, followed by cervical and lumbosacral spine.
• Treatment usually involves decompressive surgery followed by immunosuppressive treatment.

## 1 Introduction

Hypertrophic pachymeningitis (HP) is an inflammatory disorder characterized by localized or diffuse thickening of the dura mater. The estimated prevalence of this rare condition is approximately one in 100,000 individuals (Yonekawa et al., 2014). Causes include systemic inflammatory conditions, infectious diseases or tumour (Hahn et al., 2016). When such causes are excluded, it is described as idiopathic hypertrophic pachymeningitis. In the last decade, IgG4-related HP is more often recognized as one of the causes of hypertrophic pachymeningitis (Wallace et al., 2013). IgG4-related disease is an immune-mediated fibroinflammatory condition characterized by lymphoplasmacytic infiltration of IgG4-positive plasma cells, storiform fibrosis, and obliterative phlebitis (Stone et al., 2012). Hypertrophic pachymeningitis related to IgG4 can occur in absence of systemic IgG4-related disease. Therefore, histopathological analysis of dural biopsy specimens is crucial in the diagnosis of IgG4-related HP (Yang et al., 2023). Immunosuppressive therapy with glucocorticoids, steroid-sparing agents and newly monoclonal antibodies play an essential role in its treatment.

IgG4-related HP may affect the cranial or spinal dura mater or both. Depending on its localisation in the spinal canal, it can result in neurological deficits due to spinal cord or nerve root compression. Treatment usually consists of surgical decompression and thereby biopsy of the dural lesion followed by immunosuppressive treatment (Elmaci et al., 2021).

We demonstrate the case of a 45-year-old male presenting with cervical myelopathy caused by IgG4-related HSP. The clinical symptoms, radiological and histopathological findings, treatment as well as follow-up are described. Additionally, an updated review of the literature describing IgG4-related HSP is presented.

## 2 Case presentation

### 2.1 Clinical presentation

A 45-year-old male presented in an outpatient orthopaedic clinic with complaints of neck pain and numbness of the right hand. MR-imaging showing extensive thickening of the cervical meninges with absolute spinal canal stenosis and thereby spinal cord compression resulted in an emergent referral to our university hospital. On admission, the patient complained of pulsating neck pain for two months. Furthermore, he noticed fine motor skill dysfunction of the right hand as well as hypoesthesia of the right arm, right chest and contralateral hand in the last one month.

The cranial nerve examination was normal. A decrease of strength in the right arm in accordance with Medical Research Council (MRC) scale for muscle strength 4+/5 was seen with mild atrophy of the right upper arm. Symmetrical hyperreflexia with positive clonus bilaterally and positive Babinski sign on the right side were noted. Gait disturbances or bladder dysfunction were not present. Laboratory findings included high anti-neutrophil cytoplasmic antibodies (ANCA) titer of 1:160 (normal value < 1:20) without elevated levels for proteinase 3 and myeloperoxidase antibodies. Levels of IgG, IgA and IgM in blood were normal.

### 2.2 Imaging

**Fig. 1:**
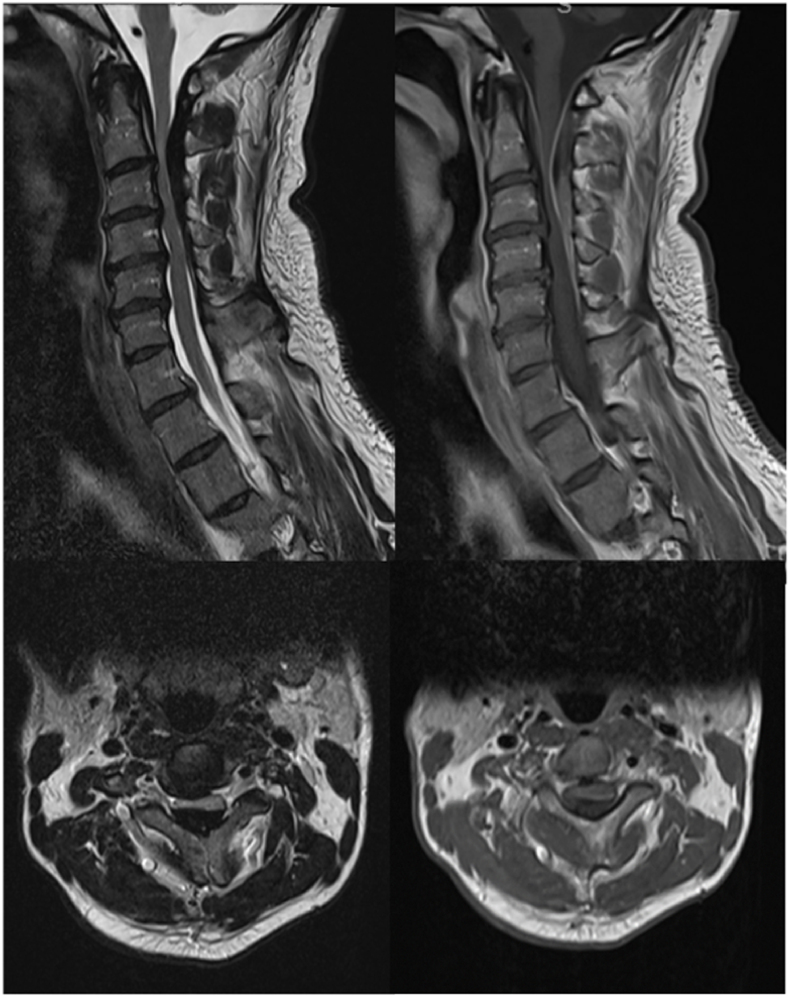
MR-imaging (T2-weighted on the left, T1 with gadolinium on the right) demonstrating thickening of the ventral and dorsal meninges from C2 up to C5 resulting in absolute spinal canal stenosis. Additional post-Gadolinium T1-weighted images show a strong enhancement of the dura mater.

Magnetic resonance imaging (MRI) of the cervical spine revealed a T1-and T2-thickening of the ventral and dorsal meninges from C2 up to C5 resulting in absolute spinal canal stenosis. Additional post-gadolinium T1-weighted images showed a strong enhancement of the dura mater. A T2-hyperintensity at the levels of C2-C3 and C3-4 as a sign of myelomalacia was present (Fig. 1). MRI of the brain showed no signs of involvement of the cranial meninges. Additional work-up including thoracic and abdominal CT showed no signs of a tumour or other abnormalities.

### 2.3 Surgery

The patient underwent surgical decompression via laminectomy C2-C5 under continuous intraoperative neuromonitoring. The dura was thickened with a hard and rubbery consistence. A large portion of the dorsal dura (3 x 2,5 × 1 cm) was microsurgically excised for decompression as well as histopathological examination. A posterior fixation using pedicle screws in C2-C4-C6 was performed. No changes in motor-evoked or sensory-evoked potentials occurred during surgery.

### 2.4 Histological examination

**Fig. 2:**
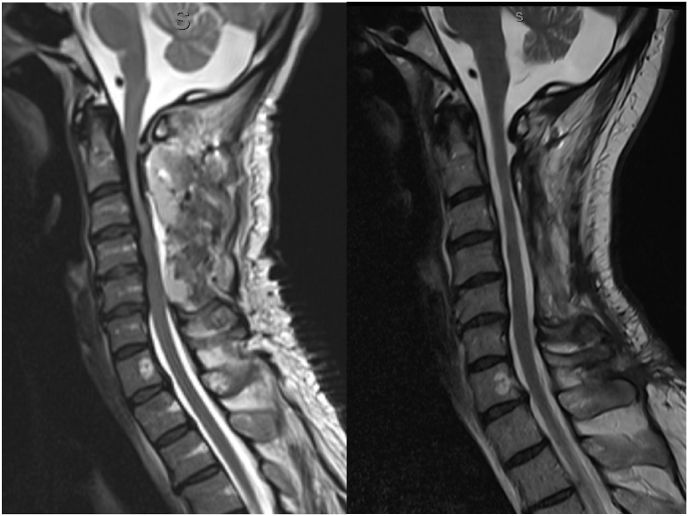
MR-imaging (T2-weighted) on third postoperative day on the left and twelve months after surgery. A regression of the T2-hypointense thickening of the dura, especially at the level C2, is noted after treatment with methylprednisolone and rituximab.

Microscopic examination of the dural specimens showed an extensive lymphocyte-dominated mononuclear cell infiltrate. Small T-lymphocytes with isomorphic chromatin-dense cell nuclei and narrow cytoplasms predominately expressing CD4 were seen. In addition, cell-dense collections of non-blastic CD20-positive B cells without conspicuously increased proliferation activity (Ki-67 index below 10%) were found disseminated in the dural tissue (Fig. 2). No evidence of an eosinophilic-granulocytic reaction was encountered. Further clonality testing with fragment length analysis of amplified regions within the variable regions of the Ig heavy chain and kappa Ig light chain gene locus yielded only polyclonal amplificates without any indication of a clonal B cell population.

### 2.5 Postoperative course

**Fig. 3:**
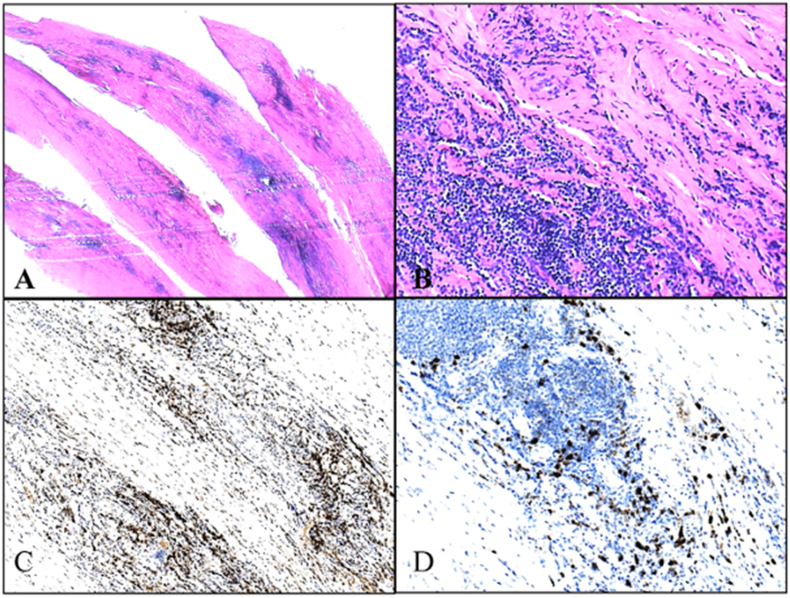
Histopathological findings. (A) Consecutive slices of thickened dura specimen showing mutifocal mononuclear inflammatory infiltrate (H&E, scale bar = 1.3 mm). (B) Dense lymphoplasmacytic cell cluster within streak of compact collagen tissue (dura mater). Note absence of granulocytes as well as multinucleated giant cells (H&E, scale bar = 90 μm). (C) The majority of lymphocytes can be classified as T4 positive/T helper cells (CD4 specific immunostaining, scale bar = 180 μm). (D) Many IgG4 positive plasma cells as part of the non-granulomatous infiltrate (IgG4 specific immunostaining, scale bar = 90 μm).

The patient reported an improvement of sensory and fine motor function of the right hand postoperatively. MRI showed an adequate decompression C3-C5 with residual stenosis at the level of C2. Due to the clinical improvement and the diagnosis of IgG4-related HSP with planed immunosuppressive treatment no second surgery was performed. Following an uneventful postoperative course, the patient received 1000mg methylprednisolone for five days followed by prednisone 10mg for four weeks. Additionally, the patient received rituximab 1000mg one, three and six months postoperatively. MRI seven months postoperatively showed no thickening of the dura and no residual spinal canal stenosis (Fig. 3).

## 3 Literature review

A systematic review was performed in accordance with the guidelines of the Preferred Reporting Items for Systematic Reviews and Meta-Analysis (PRISMA). The search was performed in the PubMed, Web of Science and EMBASE databases as per 01 Mai 2024 using the following keywords as search terms: Immunoglobulin G4-Related Disease AND pachymeningitis. Two authors (AP and CAM) independently selected the relevant publications based on the title and abstract. Only original articles in English language were selected and no restrictions in terms of publication date were applied. The two authors identified the articles that met the eligibility criteria. The inclusion of articles was based on reading the full article. Finally, the reference lists of the included articles were screened for additional publications to include. Studies were included in which patients had IgG4-related HSP either diagnosed by biopsy or CSF-analysis. The following data were extracted from the included articles: age, sex, clinical presentation, MRI characteristics, localisation of the lesion, type of immunosuppressive treatment, outcome, and follow-up.

### 3.1 Results

**Fig. 4:**
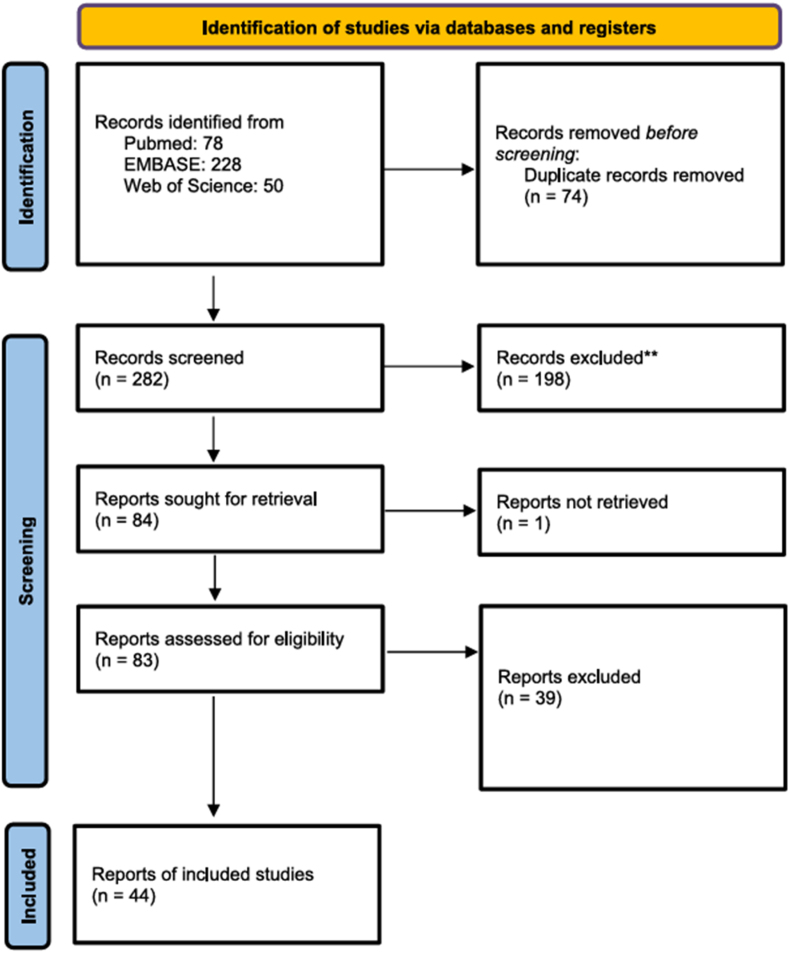
Flow diagram demonstrating the selection process of included studies.

The search syntax resulted in 78 results in PubMed, 50 results from Web of Science and 228 from the Embase database. After removal of duplicates as well as excluding by reading titles and abstracts, 84 articles were eligible for inclusion. Forty articles were excluded because of IgG4-related intracranial pachymeningitis (Fig. 4).

### 3.2 Clinical features

The literature review provided a summary of 52 cases, which included the one case from our centre (Table 1). Cervical or thoracic myelopathy was observed in 42 cases (81%). Radiculopathy was seen in two cases (4%). The lesions involved the cervical spine in 13 (25%) cases, thoracic spine in 15 (29%) cases, lumbar spine in 4 (8%) cases. In 18 (35%) cases, the lesions involved the cervical, thoracic spine and/or lumbar spine. On MR imaging, the dural lesion usually appeared T1-and T2 hypo- or isointense with strong gadolinium enhancement. Decompressive surgery was performed in 33 cases (63%), whereas the surgical treatment was not mentioned in 7 (13%) cases. The treatment was followed by immunosuppressive treatment in almost all well-described cases with glucocorticoids being the treatment of choice. Rituximab was given in 6 cases (12%) without any signs of relapse. The follow-up period varied from 0 months up to 60 months.

**Table 1:** List of cases of IgG4-HSP.

Article number	Author and Year	Age and Sex	Clinical Presentation	Location of the lesion	Systemic, cranial or other organ involvement	Radiological findings	Surgery	Steroid therapy	Rituximab	Other immunosuppressive drugs	Outcome	Follow-up period (months)
1	Chan et al., 2009	37 M	Bilateral lower limbs weakness, T9 sensory level deficits, unsteady gait	T5-T10	Bilateral submandibular gland involvement.	STIR: hyperintense. T1 Post-contrast: homogeneous enhancement.	Decompressive surgery	N/A	N/A	N/A	N/A	N/A
2	Choi et al., 2010	46 F	Progressive bilateral weakness and sensory deficit in the lower limbs.	T9-T11	None	T2WI: hypointense. T1 Post-contrast: high enhancement.	Decompressive surgery	Yes	N/A	Anti-tuberculosis drugs	Improved	8
3	Lindstrom et al., 2010	55 M 63 M	Spinal cord compression Bilateral hand numbness	C3-C7 C2-C3	N/A N/A	N/A N/A	Unspecified Unspecified	Yes N/A	N/A N/A	N/A N/A	Improved N/A	15 N/A
4	Della Torre et al., 2012	65 M	Headaches with dysarthria	Posterior cranial fossa to C4	Cranial dura and abdominal periaortitis.	T1 Post-contrast: high enhancement.	Meningeal biopsy	Yes	N/A	Methotrexate and Cyclophosphamide	Improved	12
5	Tajima et al., 2012	64 M	Dysphagia, hoarseness, atrophy of the tongue and sternocleidomastoid muscle on the right side.	T3-T11	Cranial dura and renal involvement.	N/A	Open biopsy of the thoracic lesion	Good	N/A	N/A	Improved	0.7
6	Wallace et al., 2013	32 M	Radicular sensory deficit and weakness.	L5	N/A	N/A	Decompressive surgery	N/A	N/A	N/A	N/A	N/A
7	Della-Torre et al., 2013	48 F	Left facial pain and palsy with bilateral sensorineural hearing loss and dysphagia.	Cranial dura to C1	Cranial dura and orbital involvement.	T1 Post-contrast: high enhancement.	Meningeal biopsy	Good	N/A	Cyclophosphamide	Improved	8
8	Kim et al., 2014	52 F	Sudden bilateral lower limbs weakness, T5 sensory level deficits and bladder dysfunction.	C7 - T5	None	T2WI: hypointense. T1 Post-contrast: homogeneous enhancement.	Decompressive surgery	Poor	N/A	N/A	Improved	2
9	Sakai et al., 2014	32 F	Visual disturbances, diabetes insipidus, neck pain and lower limbs weakness bilaterally.	C1-C7 and L5-S1	Cranial dura, pituitary and lungs involvement.	T1 Post-contrast: high enhancement.	Meningeal biopsy	Good	N/A	N/A	Improved	6
10	Zwicker et al., 2014	58 F	Periscapular pain with bilateral lower limbs weakness and sensory deficits.	C7-T6	None	T1WI: hypointense. T2WI: hypointense. T1 Post-contrast: high enhancement.	Decompressive surgery	Good	N/A	Methotrexate	Improved	27
11	Chen et al., 2014	49 M	Thoracic back pain and right lower limb sensory deficit.	T1-T3 L4-L5	None	T1 Post-contrast: high enhancement.	Laminectomy (STR)	Good	N/A	Anti-tuberculosis drugs	Improved	16
12	Ezzeldin et al., 2014	55 M	T4/T5 sensory level deficits and paraplegia.	T2-T3	N/A	T2WI: hypointense. T1 Post-contrast: homogeneous enhancement.	Biopsy followed by decompressive surgery	Good	N/A	N/A	Improved	N/A
13	Gu et al., 2016	43 M	Neck pain, thoracic myelopathy with bowel and bladder dysfunction followed by cervical myelopathy.	C4-T2	Not done	T1WI: hypointense. T2WI: hyperintense.	Decompressive surgery	Good	N/A	N/A	Improved	6
14	Lu et al., 2016	55 M	Quadriparesis and sensory deficits followed by 7 days of obstructed defecation and dysuria.	C2-T9	None	T1 Post-contrast: homogeneous enhancement.	Decompressive surgery	Good	N/A	cyclophosphamide	Improved	8
15	Ferreira et al., 2016	57 F	Back pain for 2.5 years, severe progressive paraparesis, gait disturbance and sensory loss.	T10-T12	None	T1 Post-contrast: homogeneous enhancement.	Decompressive surgery	Good	N/A	N/A	Improved	6
16	Radotra et al., 2016	50 M 19 M	Progressive bilateral lower limbs weakness and wasting with difficulty voiding. Back pain with left lower limb radicular pain and weakness.	L1–2 L2-L3	N/A N/A	T1WI: isointense. T2WI: hypointense. T1 Post-contrast: homogeneous enhancement. T1WI: isointense. T2WI: hypointense. T1 Post-contrast: homogeneous enhancement.	Decompressive Surgery Biopsy	Yes Yes	N/A N/A	N/A N/A	Improved Improved	7 6
17	Yangue et al., 2016	18 M	Weakness and sensory deficits.	C1-C7	N/A	N/A	Decompressive surgery	N/A	N/A	N/A	Improved	N/A
18	Zhao et al., 2017	49 F	Right upper back pain.	T1–T4 .	None	T1WI: hyperintense. T2WI: hyperintense. T1 Post-contrast: homogeneous enhancement.	Decompressive surgery	N/A	N/A	N/A	N/A	N/A
19	Fernández-Codina et al., 2017	55 M	Headaches, horizontal gaze impairment, dysphagia and scapular palsy.	Cranial dura up to C3	Cranial dura, pleura and lungs	T1 Post-contrast: homogeneous enhancement.	Pleuro-pulmonary biopsy	Poor	Yes	N/A	Improved	10
20	Williams et al., 2017	46 F	Neck pain with weakness and sensory deficits in both upper limbs.	C4-T1	None	T1WI: hypointense. T1 Post-contrast: homogeneous enhancement.	Biopsy	Good	N/A	Azathioprine	Improved	6
21	Maher et al., 2017	79 F	Right-sided thoracic back pain.	C6- L2	N/A	T1WI: hypointense. T2WI: hypointense. T1 Post-contrast: homogeneous enhancement.	Decompressive surgery	Poor	Yes	N/A	Died from infection	4.5
22	Bridges et al., 2019	68 M	Intermittent thoracic pain for 3 years, disequilibrium, progressive trunk numbness and bilateral lower limbs numbness for 6 months.	T3-T5	None	T1WI: hypointense. T2WI: hypointense. T1 Post-contrast: homogeneous enhancement.	Decompressive surgery	Good	N/A	N/A	Improved	3
23	Rumalla et al., 2017	50 M	Back pain for 3 months, acute onset of paraplegia, T6 sensory level deficits and urinary retention.	T4-T6	Lungs	T1 Post-contrast: homogeneous enhancement.	Decompressive surgery	Good	N/A	N/A	Improved	6
24	Varrassi et al., 2018	62 M	Paresthesia, burning dysesthesia, severe hyposthenia and severe muscular atrophy of both lower limbs.	C4 -T1	Cranial dura and orbital involvement.	T1WI: hypointense. T2WI: hyperintense. T1 Post-contrast: homogeneous enhancement.	Orbital biopsy	Poor	Yes	N/A	Improved	N/A
25	Winkel et al., 2018	48 F	Lower back pain, neurogenic claudication and right lower limb radiculopathy.	L2-L3	None	T2WI: hypointense. T1 Post-contrast: homogeneous enhancement.	Biopsy	Good	N/A	N/A	Improved	12
26	Cação et al., 2019	56 F	Medullary symptoms and lumbar pain.	Thoracic and lumbar spine	None	T2WI: hypointense. T1 Post-contrast: homogeneous enhancement.	N/A	Good	N/A	N/A	Improved	N/A
27	Levraut et al., 2019	55 M	Progressive walking difficulty, severe paresis, thoracic back pain, sensory deficit in both lower limbs and bladder dysfunction.	C3-T3	None	T1WI: isointense. T2WI: hypointense. T1 Post-contrast: homogeneous enhancement.	Decompressive surgery	Good	N/A	N/A	Improved	3
28	Merza et al., 2019	60 F	Weakness and numbness in all four limbs with bowel and bladder incontinence.	T1-T7	None	T1 Post-contrast: homogeneous enhancement.	Decompressive surgery	Good	N/A	N/A	Improved	60
29	Sireesha et al., 2019	40 F	Compressive myelopathy.	C5-T6	None	Spinal cord involvement extending from C5 to T6	Biopsy	Good	N/A	N/A	Improved	3
30	Slade et al., 2019	50 M	Lower limbs hypesthsia and subjective weakness, upper back pain for 2 months and ataxia for 3 months.	C7-T5	N/A	T2WI: hypointense.	Decompressive surgery	Poor	Yes	N/A	Improved	26.5
31	Melenotte et al., 2019	57M 57M 68 M 31M	Cervicobrachial palsy Medullary syndrome Paraparesis with lumbar back pain. Headaches with cervicalgia	Cervical spine T2-T3 T9 - L2 C3 - C5	None None None Cranial dura and orbital involvement.	T1 Post-contrast: homogeneous enhancement. A dural lesion with medullar compression on T2-T3 Epidural thickening from T9 to L2 with medullar compression. T1 Post-contrast: homogeneous enhancement.	Unspecified Unspecified Unspecified Unspecified	Yes N/A N/A Yes	N/A N/A Yes N/A	N/A N/A N/A N/A	Improved Improved Improved Improved	126 15 20 17
32	Sbeih et al., 2020	24 M	Back pain for 4 years, heaviness and numbness in the lower limbs followed by walking difficulty.	C7 - T6	None	T1WI: hypointense. T2WI: hypointense. T1 Post-contrast: homogeneous enhancement.	Decompressive surgery	Good	N/A	N/A	Improved	65
33	Vakrakou et al., 2020	17 F	Myelopathy	C2-C5	Deep white matter and hypophysis involvement	T2 hyperintense	Biopsy	Good	N/A	Azathioprine	Improved	60
34	Li et al., 2020	58 F	Back pain	T2-T7	None	T1 and T2 hypointense with T1 Post-contrast: homogeneous enhancement.	Decompressive surgery	Good	N/A	Cyclophosphamide	Improved	9 ✝
35	Elmaci et al., 2020 (Elmaci et al., 2021)	37 F	Neck pain with numbness of hands	C2-T3	None	T1 and T2 hypointense with T1 Post-contrast: homogeneous enhancement.	Decompressive surgery	N/A	N/A	N/A	Improved	24
36	Woo et al., 2021	62 M	Neck pain with right sided weakness	C4-C6	None	T1 and T2 hypointense with T1 Post-contrast: homogeneous enhancement.	Decompressive surgery	Good	N/A	Azathioprine	N/A	2
37	Sharma et al., 2021	27 M	Numbness and weakness in all extremities	C2-C4	Cranial involvement	T1 and T2 hypointense with T1 Post-contrast: homogeneous enhancement.	Decompressive surgery	Poor	Yes	N/A	Improved	11
38	Sankowski et al., 2021	56 M	Ataxia	Foramen magnum to C7	None	T1 and T2 hypointense with T1 Post-contrast: enhancement.	Biopsy	Yes	N/A	N/A	N/A	N/A
39	Yang et al., 2023	68 M 43 M 39 M	Paraplegia with MRC 0 Sensory level T12, Paraplegia with involvement of Triceps muscles Sensory level T12, Paraparesis	T9-T11 C4-T2 C5-T4	None None None	T1 and T2 hypointense with T1 Post-contrast: enhancement. T1 hypointense, T2 slightly hyperintense with T1 Post-contrast: enhancement. T1 hypointense, T2 hyperintense with T1 Post-contrast: enhancement.	Decompressive surgery Decompressive surgery Decompressive surgery	Yes Yes Yes	N/A N/A N/A	N/A N/A N/A	Improved Improved Improved	6 6 5
40	Nathan et al., 2023	41 F	Paraplegia	T12	None	Isointense to the cord on T1, T2, and STIR imaging	Decompressive surgery	N/A	N/A	N/A	Improved	6
41	Araújo et al., 2023	40 F	Cervical myelopathy	Cervical, thoracic and lumbar	None	T1 and T2 hypointense with T1 Post-contrast: enhancement.	Decompressive surgery	Bad	Yes	Azathioprine	Improved	60
42	Gader et al., 2023	25 M	Thoracic myelopathy and back pain	T5-T10	None	T1 isotense, T2 slightly hyperintense, T1 Post-contrast: enhancement.	Decompressive surgery	N/A	N/A	N/A	Improved	N/A
43	Srinivasa Chennareddy et al., 2023	57 M	Thoracic myelopathy and back pain	T5-T7	None	T1 and T2 hypointense with T1 Post-contrast: striated appereance.	Decompressive surgery	Good	Yes	N/A	Improved	12
44	Yu et al., 2023	55 F	Back pain and thoracic myelopathy	Cervical, thoracic and lumbar	None	T1 Post-contrast: enhancement.	Decompressive surgery	Poor	N/A	N/A	Improved	6
45	Actual report	45 M	Neck pain and cervical myelopathy	C2-C5	None	T1 and T2 hypointense with T1 Post-contrast: enhancement.	Decompressive surgery	Good	Yes	N/A	Improved	12

## 4 Discussion

IgG4-related hypertrophic spinal pachymeningitis is a rare cause of spinal cord and nerve root compression. The most frequent localisation described in the literature is the thoracic spine, followed by the cervical spine. Consequently, myelopathy is the most common clinical symptom at first presentation (81%). Systemic IgG4-related disease as well as intracranial involvement were found in 21% of the cases, resulting in isolated spinal affection as the most common entity. In most cases, MR imaging of IgG4-related HSP usually demonstrates hypointense signal on T1-and T2-weighted images with strong enhancement on gadolinium-enhanced T1-weighted images. However, localised thickening mimicking meningeomas can also occur.

In contrast to cranial involvement, surgery seems to play a crucial role in the treatment of IgG4-related HSP, as many patients present with severe compression of neurogenic structures. Surgery allows for decompression and biopsy of the dural lesion allowing for accurate diagnosis. Intraoperatively, the dura is often described as thickened, rubbery, firm in consistency, greyish in colour and moderately vascular (Sbeih et al., 2020). In our case, we found the dura having the same aspect as described in the literature.

Immunosuppressive therapy plays a key role in the treatment of IgG4-related HP (Lu et al., 2016). Glucocorticoids are most described in the literature. Other alternatives include cyclophosphamide, aziothioprine and recently rituximab. Rituximab is a monoclonal antibody that targets CD20 and results B-cell depletion. It has been described as an effective treatment in IgG4-related disease and has also been used in the treatment of IgG4-related HSP. To our knowledge, no recurrence has been described in patients with IgG4-related HSP treatment with rituximab, suggesting it may be the current treatment of choice.

The pachymeningeal thickening seen in IgG4-related HSP may resolve after immunosuppressive treatment. In the presented case, a residual dural thickening was present at C2-level but resolved after medical treatment. In our opinion, if IgG4-related HSP is suspected, surgery should aim at decompressing the spinal cord at the level of severe stenosis caused by the hypertrophic dura and not total resection of it. This allows for histopathological diagnosis and minimizes any surgical complications.

## 5 Limitations and conclusions

There are several limitations to this study. The retrospective and descriptive nature of this study makes generalizability unfeasible. The small cohort of patients makes it difficult to draw conclusions regarding optimal treatment. Moreover, there is a heterogeneity in surgical treatment as well as immunosuppressive treatment. In our opinion, surgery including bony decompression as well as dural excision plays an essential role in the initial treatment of decompressing the spinal cord. The subsequent immunosuppressive therapy is the cornerstone treatment of IgG4-related HSP.

## Author contributions

AP: Conceptualization, Data collection, Analysis, Writing.

CAM: Data collection, Interpretation, Revision.

TPS, UB and HC: Revising.

KWN: Analysis, Writing.

CB: Conceptualization, Analysis, Drafting, Revision and Approval.

All authors have read and agreed to the published version of the manuscript.

## Funding

This research received no external funding.

## Consent to publication

The patient, whose case is described in this paper, has consented to the description of his/her case as well as to the publication of the radiological and intraoperative images.

## Declaration of competing interest

The authors declare no conflict of interest.

## Abbreviations:

IgG4-RD: Immunoglobin G4-related disease
MRI: Magnetic resonance imaging
HP: Hypertrophic pachymeningitis
HSP: Hypertrophic spinal pachymeningitis
ANCA: Anti-neutrophil cytoplasmic antibodies
MRC: Medical Research Council
